# Environmental and Physiological Factors Associated With Stamina in Dogs Exercising in High Ambient Temperatures

**DOI:** 10.3389/fvets.2017.00144

**Published:** 2017-09-11

**Authors:** Patrick J. Robbins, Meghan T. Ramos, Brian M. Zanghi, Cynthia M. Otto

**Affiliations:** ^1^Penn Vet Working Dog Center, University of Pennsylvania School of Veterinary Medicine, Philadelphia, PA, United States; ^2^Nestlé Purina Research, St. Louis, MO, United States

**Keywords:** stamina, exercise, detection dogs, core body temperature, outdoor temperature, blood gas

## Abstract

This IACUC approved study was performed to evaluate the environmental, physiological, and hematological components that contribute to stamina following successive bouts of exercise that included searching (5-min), agility (5-min), and ball retrieve (<10-min). Regularly exercised dogs (*N* = 12) were evaluated on five separate occasions. The population consisted of eight males and four females ranging in age from 8 to 23 months, which included six Labrador retrievers, three German shepherds, and one each English springer spaniel, German wirehaired pointer, and Dutch shepherd. The exercise period was up to 30 min with 5 min of intermittent rest between the exercise bouts or until a designated trainer determined that the dog appeared fatigued (e.g., curled tongue while panting, seeking shade, or voluntary reluctance to retrieve). At the end of the exercise period, pulse rate (PR), core temperature, blood lactate, and venous blood gas were collected. The median outdoor temperature was 28.9°C (84°F) (IQR; 27.2–30°C/81–86°F) and median humidity was 47% (IQR; 40–57%). Median duration of exercise was 27 min (IQR; 25–29). No dog showed signs of heat stress that required medical intervention. The components used to measure stamina in this study were total activity, post-exercise core body temperature (CBT), and increase in CBT. When controlling for breed, total activity, as measured by omnidirectional accelerometer device, could be predicted from a linear combination of the independent variables: pre-exercise activity (*p* = 0.008), post-exercise activity (*p* < 0.001), outdoor temperature (*p* = 0.005), reduction in base excess in extracellular fluid compartment (BEecf) (*p* = 0.044), and decrease in TCO_2_ (*p* = 0.005). When controlling for breed and sex, increase in CBT could be predicted from a linear combination of the independent variables: study day (*p* = 0.005), increase in PR (*p* < 0.001), increase in lactate (*p* = 0.001), reduction in BEecf (*p* = 0.031), increase in glucose (*p* = 0.044), increase in hematocrit (*p* = 0.032), and increase in hemoglobin (*p* = 0.038). This study suggests that the influence of outdoor temperature, pre- and post-exercise activity, and the metabolic parameters are important components of stamina associated with exertion.

## Introduction

Exercise, conditioning, and physical fitness are important to both working and active pet dogs. For working dogs to reach a level of field readiness, it is essential that the dog achieves and maintains top athletic performance (1–4), which includes fitness level. Working dog fitness is a combination of cardiorespiratory function, balance, strength, flexibility, proprioception, stamina, and muscle strength. Stamina is the ability to withstand high energy-demanding activity over extended periods of time. Many factors may influence or even limit stamina in dogs. These can include intrinsic factors of the athlete, such as muscular activity, fat and electrolyte metabolism, body weight and conditioning, extrinsic factors such as environmental temperature and humidity, and fixed factors including age, breed, and sex (2–4). Additional factors including acclimatization to environment and activity, such as exercise conditioning, to increase fitness may also play a role in increasing stamina in dogs.

As athletes, dogs have been used to study the multi-systemic effects of exercise and factors that limit performance. During exercise, dogs exert energy that leads to heat generation (5–7). Canine athletes have a higher cardiovascular and thermoregulatory demand that requires sport and working dogs to have a greater internal temperature and cardiac regulation. During exercise, the dog is capable of increasing its cardiac output by 74–200% (7–13) and increases its carotid blood flow by 500% (14–16), which can increase blood flow to regions of maximal heat exchange perhaps as a strategy for heat tolerance.

Temperature regulation in dogs is primarily a function of respiratory exchange and associated evaporative heat loss (17, 18). The combination of exercise and limited evaporative cooling through panting leads to several physiological changes, which include tachypnea, lactic acidosis, respiratory alkalosis, hyperthermia, increase in heart rate, and hypocapnia (2–4, 6–8, 13, 19–28). When the magnitude of these shifts occurs beyond the physical capacity or conditioning of the dog, its ability to work for prolonged durations is limited and the need for frequent rest periods is required.

Even with the existing body of research, the most important factors in limiting canine stamina have not been fully characterized.

This study evaluated the environmental, physiological, dog-specific variables that influence exercise stamina in regularly exercised dogs. We hypothesized that canine stamina (defined as a two-component assessment of activity, as measured by an accelerometer and duration of exercise) would be primarily limited by the external factors of environmental temperature and humidity. If external factors are the main predictors of exercise stamina, then strategies to increase stamina will need to focus on managing those factors or adjusting expectations of stamina with the changes in heat and humidity. By contrast, if dog-specific factors are the main predictors of stamina then training or conditioning may provide a mechanism to increase activity counts per duration of exercise.

## Materials and Methods

### Animal Care and Feeding

The current study protocol (#805342) was approved by the University of Pennsylvania Institutional Animal Care and Use Committee. Twelve regularly exercised dogs (eight males and four females) of five different breeds; six Labrador retrievers, three German shepherd dogs, one Dutch shepherd dog, one Springer spaniel, and one German wirehaired pointer were tested (Table 1). Breed was assigned as a categorical variable for our analysis. Age of the dogs ranged from 8 to 23 months (median, 16 months). The dogs’ weights ranged from 31.7 to 80.0 lbs (14.4–36.4 kg). All dogs used in the exercise trial were required to have overall good general health prior to beginning the trial and were evaluated by a veterinarian at the beginning of each trial week. All dogs participating in the study had been previously trained to retrieve and perform various agility/search tasks. Exercise conditioning was defined as daily exercise of similar type and duration as used in the exercise protocol for at least 4 weeks preceding the study. Dogs were owned by the University of Pennsylvania and enrolled in the Penn Vet Working Dog Center (PVWDC) training program where each dog trains daily and lived in a foster home during evenings and weekends. Dogs were individually housed in a metal wire crate at the PVWDC when not training or exercising during the day. All field exercise and sample collection were conducted on the grounds of the PVWDC.

**Table 1:** Information on study population.

Dog name	Breed	Age	Sex
Ohlin	Labrador Retriever (LaR)	23	M
Tsunami	German Shepherd	22	F
Sirius	LaR	24	M
Ffoster	LaR	42	F
Gus	LaR	14	M
Quest	German Shepherd	8	M
McBaine	Springer Spaniel	21	M
PApa Bear	LaR	25	M
Ditto	German Wired Haired Pointer	16	M
Pacy	LaR	16	F
Logan	German Shepherd	8	M
Felony	Dutch Shepherd	7	F

All dogs were fed either two or three times daily with *ad libitum* water to maintain an optimal body condition score between 4 and 5 out of 9 (29). Dogs were maintained on commercial dry kibble (Purina Pro Plan Sport All Life Stages Performance 30/20 Formula Dog Food or Pro Plan Focus Sensitive Skin and Stomach Formula Dog Food, Nestlé Purina PetCare Company, St. Louis, MO, USA) through the duration of the trial. Dogs were given *ad libitum* water before the exercise challenge and after the 5-min post-exercise measurements. For dogs fed three times daily, the mid-day feeding was delayed until after all post-exercise measurements were taken.

### Experimental Design and Exercise

**Figure 1:**
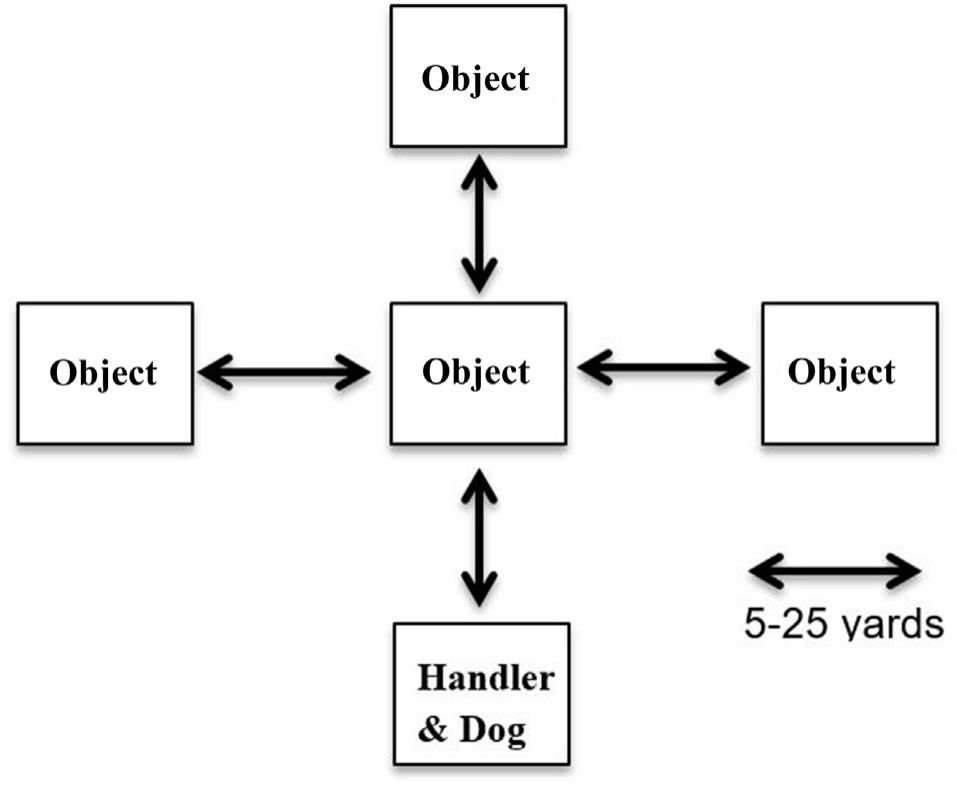
Direction and control training is used to train disaster search dogs to safely navigate or avoid hazardous areas. Modeled after a baseball diamond, handler can direct the dog to obstacles behind, in front of, to the left or to the right of, the dog (distances ranging from 5 to 25 yards).

**Figure 2:**
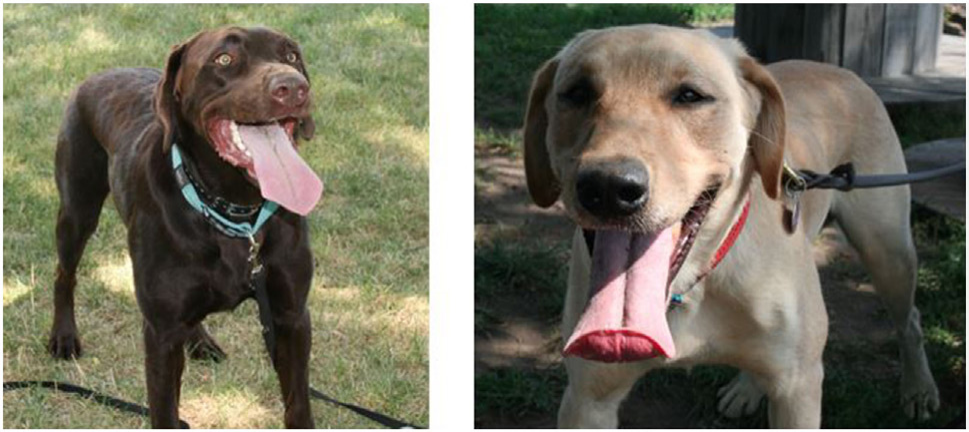
Dogs during and after exercise demonstrating signs of exercise in the heart, include spade tongue, squinty eyes, ears retracted.

The exercise field study was designed to assess a 30-min exercise challenge conducted on five separate days over a 19-day period (days 1, 7, 12, 15, and 19). The exercise challenge was conducted to assess the effect of exercise in warm ambient temperatures in a setting that reproduced various types of physical activity (scent searching/tracking, agility, and retrieving) typical of training activities for detection dogs. The study consisted of two study periods with six different dogs randomly assigned to participate in each period. The first experimental period occurred between June 12 and July 1, 2014, whereas the second was between August 7 and August 25, 2014. Temperature and humidity were measured using a wireless device (AcuRite Wireless Weather Station; Primex Family of Companies, Lake Geneva, WI, USA), which was placed at the site of the outdoor exercise challenge on each study day. After an initial pre-exercise activity of trotting, accompanied by active stretches for 5 min, the outdoor exercise challenge was designed to be ≤30 min long; consisting of 5 min of search, followed by 5 min of rest in the shade, then 5 min of agility, followed by 5 min of rest in the shade, and a maximum 10 min of ball retrieve, and then a post-exercise activity period of light trotting/quick walking for 5 min. This duration of exercise is similar to previous studies evaluating exercise-induced physiological changes (3, 4, 28). The start time of each exercise component was documented manually. The search component of the exercise challenge consisted of each dog locating and alerting on 2–3 hidden scent sources (live humans or human cadaver remains) in a simulated rubble pile. Human cadaver remains samples was provided by Sarah Atlas of the New Jersey Task Force, who gave the sample to the PVWDC Training Manager, who is also a Canine Search Specialist with a certified Federal Emergency Management Agency (FEMA) Urban Search and Rescue Human Remains Detection Dog. The sample was 18 g of knee tissue. The sample was received on June 1, 2014 and was a 1-month old sample. The agility portion consisted of dogs climbing elevated vertical and horizontal ladders angled 0–45°, walking over unstable surfaces, cavalettis, performing distance exercises (e.g., direction and control) (Figure 1), and moving through tunnels, in a controlled pace set by the trainer. Following success in agility or search, dogs were rewarded with tug or ball play. For the rest components, dogs were held on leash by a stationary trainer. Dogs were allowed to sit, stand, or walk within the six-foot length of their leash. The ball-retrieve component consisted of dogs retrieving a ball or toy. Dogs were allowed up to 15 s between retrieves. Because some dogs (*n* = 2) of different breeds did not naturally retrieve, they were trotted on leash in a ball-retrieve fashion until exercise was stopped. Dogs were allotted 15 s between trots. Each exercise portion (search, rest, agility, and ball retrieve) occurred in the same designated location and order for each dog in each trial. The exercise test was stopped if dogs showed signs of curled tongue while panting, seeking shade, or slowing of retrieve (Figure 2). To keep the exercise termination consistent among dogs, a single experienced canine trainer (the PVWDC training manager) determined the end of exercise in each trial.

The exercise challenge started at 12:00 p.m. each day for moderate external ambient temperature challenge on the five sample collection days for each experimental period. The order of dogs in the exercise challenge was randomized on each sample collection day, except when medical reasons dictated otherwise. Tracheobronchitis, with no signs of fever or systemic illness, was first recognized in one dog on August 18th. On August 19th, two more dogs were also observed coughing. By August 26th, all but two dogs were observed with a non-productive cough.

### Sample Collection and Physiological or Biochemical Measurements

Pulse rate (PR), left (LET) and right (RET) ear temperatures, and core body temperature (CBT) were recorded for each dog on each of the five exercise challenge days either between 7 and 9 a.m. (pre-exercise), and immediately after exercise. Manual PR was obtained from the femoral artery. Left and right ear temperatures were collected using an instant ear thermometer device (Pet-Temp^®^ Instant Infrared Ear Thermometer for Home Use, Model PT-300, San Diego, CA, USA) (30). Body temperature was collected using a telemetric core temperature monitor (CorTemp^®^ Indigestible Core Body Temperature Sensor, HQInc., Palmetto, FL, USA) to measure CBT within the gastrointestinal tract (CBT). New CorTemp sensors were swallowed by the dogs the morning of each exercise challenge day. CBT data measured by the CorTemp sensor was collected wirelessly by the CorTemp Data Recorder (HQInc., Palmetto, FL, USA).

Similar to previous studies, the following data were collected (2–4, 6–8, 13, 19–28). Two samples of venous blood (~3 mL; jugular, cephalic, or saphenous) were collected on each exercise challenge day to evaluate pre- and immediate post-exercise analytes. Pre-exercise blood samples were collected between 7 and 9 a.m., whereas post-exercise blood samples were collected immediately (0-min) post-exercise before the cooldown period. Blood samples were taken anaerobically from a peripheral vein and analyzed immediately. Venous blood pH, gases (PvCO_2_, HCO_3_, TCO_2_, PvO_2_, SvO_2_), electrolytes (iCa, Na, K), base excess in extracellular fluid compartment (BEecf), glucose, hemoglobin, and hematocrit using point of care i-STAT CG8 + cartridge (Abaxis, Union City, CA, USA) were analyzed using a VetScan i-STAT 1 Handheld Analyzer (Abaxis, Union City, CA, USA). Blood lactate was measured using point of care analysis (Lactate Scout+, EKF Diagnostics GmbH, Magdeburg, Germany).

### Locomotor Activity

Locomotor activity was measured using an omnidirectional accelerometer device (version 3.1, Actical^®^, Respironics, Koninklijke Philips Electronics, Bend, OR, USA) placed inside a specially designed case and attached to the dog’s collar between 8 and 10 a.m., and taken off after final post-exercise 60-min measurements. The activity device was set to record activity counts with a 1-min epoch. The total activity data encompassed the warm up (5-min period of trotting/stretching before start of search exercise), exercise period, and the cooldown (5-min period of trotting/stretching after end of ball-retrieve exercise). The activity data files were batch processed using the Actical software (version 3.1, Actical^®^, Respironics, Koninklijke Philips Electronics, Bend, OR, USA). All epoch data were consolidated into a single Microsoft Excel file for alignment of activity count time-stamp with the manually recorded time of exercise.

### Statistics

A descriptive and normality test (Kolmogorov–Smirnov) was used to determine if the data were normally distributed. Backwards-stepwise regressions were performed with stamina and change-from-baseline increase in CBT as the dependent variables; the independent variables included environmental, physiological, and hematological measurements taken for each dog on each trial day. A two-way ANOVA was used to test the effect of tracheobronchitis on post-exercise CBT and stamina. A Mann–Whitney Ranked Sum test was performed to measure the differences of temperature and humidity between the two experimental time periods dogs were tested in. Breed and gender were entered as categorical values. A linear mixed model was run where Time, Day, and Time × Day were entered in as fixed effects and animal ID was entered as a random effect. The *p-*values of least square means in Tables 2 and 3 were generated from the linear mixed model.

**Table 2:** Environmental and exercise measurements.

	Mean ± SE	Median; IQR
Temp (°C/°F)	28.7 ± 0.7/83.7 ± 0.7	28.9; 27.2/84; 81–86
Humidity (%)	49.6 ± 1.5	47; 40–57
Duration of exercise (min)	27.1 ± 0.3	27; 25–29
Average activity counts/min	1,807.8 ± 52.1	1,782.1; 1,498.4–2,028.9

**Table 3:** Pre- and post-exercise physiological measurements.

	Pre-exercise		Post-exercise		*p*-Value
	Mean ± SE	Median; IQR	Mean ± SE	Median; IQR	
Pulse rate, beats/min	86.9 ± 3.3	84; 78–96	134.6 ± 3.3	132; 120–152	<0.001
Core body temperature, °C/°F	38.3 ± 0.3/101.0 ± 0.3	38.5; 38.3 38.8/101.3; 100.9–101.8	40.1 ± 0.1/105.4 ± 0.3	40.7; 40.3–41.4/105.3; 104.6–106.5	<0.001
Left ear temperature, °C/°F	37.3 ± 0.1/98.8 ± 0.4	37.2; 36.8–37.7/98.9; 98.2–99.9	39.8 ± 0.1/103.7 ± 0.4	39.9; 39.1–40.8/103.8; 102.3–105.4	<0.001
Right ear temperature, °C/°F	37.2 ± 0.2/98.9 ± 0.4	37.1; 36.8–37.7/98.8; 98.3–99.9	39.9 ± 0.1/103.9 ± 0.3	39.9; 39.1–40.8/103.8; 102.2–105.4	<0.001

## Results

### Baseline and Post-exercise Physiological and Hematological Measures

Over the two experimental periods, outdoor temperature averaged 28.7 ± 0.7°C/83.7 ± 0.7°F, and outdoor humidity averaged 49.6 ± 1.5%. Mean duration of exercise was 27.1 min. There was no significant difference in outdoor temperature between the first (June 12–July 1, 2014) (median 29.2°C/84.5°F) and second (August 7–August 25, 2014) (median 28.9°C/84°F) experimental time periods (*p* = 0.17). There was a significant difference in humidity between the first (median 50%) and second (median 41%) experimental time periods (*p* = 0.02). Stamina, as measured by omnidirectional accelerometer device, reported 1,782.1 (1,498.4–2,028.9); total exercise activity counts per min epoch (median; IQR). Environmental and exercise measurements combined for the two experimental periods are shown in Table 2.

All dogs completed each trial day of the study. All pre-exercise baseline blood values were within reference ranges. Means and medians of pre- and post-exercise physiological measurements are listed in Table 3, whereas, means and medians of pre- and post-exercise hematological measurements are listed in Table 4. CBT, LET, RET, PR, pH, partial pressure of oxygen (PvO_2_), oxygen saturation in peripheral venous blood (SvO_2_), glucose, hematocrit, and hemoglobin all increased (*p* < 0.001) after exercise compared to pre-exercise baseline measurements. PvO_2_, BEecf, bicarbonate (${\text{HCO}}_{3}^{-}$), total carbon dioxide (TCO_2_), and ionized calcium (iCal) decreased (*p* < 0.001) after exercise compared to pre-exercise baseline measurements. Mean change in pre- and post-exercise CBT was +1.8°C (4.4°F). Mean change in pre- and post-exercise LET was +2.5°C (4.9°F) and RET was +2.7°C (5.0°F).

**Table 4:** Pre- and post-exercise hematological measurements.

	Pre-exercise		Post-exercise		*p*-Value
	Mean ± SE	Median; IQR	Mean ± SE	Median; IQR	
Lactate reading, mmol/L	1.2 ± 0.09	1.2; 1.0–1.4	1.4 ± 0.09	1.3; 1.0–1.7	0.090
pH	7.37 ± 0.02	7.37; 7.35–7.39	7.56 ± 0.02	7.54; 7.49–7.66	<0.001
PvCO_2_, mmHg	37.5 ± 1.06	37.2; 34.2–40.2	20.0 ± 1.06	19.5; 15.1–24.5	<0.001
PvO_2_, mmHg	45.4 ± 4.03	44; 34–52	70.4 ± 4.03	59; 46–78	<0.001
Base excess in extracellular fluid compartment, mmHg	−3.6 ± 0.48	(−)3.4; (−)5–(−)2	−4.8 ± 0.48	(−)5; (−)6.75–(−)3	<0.001
HCO_3_, mmHg	21.5 ± 0.43	21.4; 19.7–23	17.3 ± 0.43	17.1; 15.9–18.7	<0.001
TCO_2_, mmHg	22.8 ± 0.47	23; 21–25	17.8 ± 0.47	17; 16–19	<0.001
SvO_2_, %	74.0 ± 2.2	78.5; 64–85	92.4 ± 2.2	94; 90–98	<0.001
Sodium, mmol/L	145.2 ± 0.32	145; 144–146	145.0 ± 0.32	145; 144–146	<0.001
Potassium, mmol/L	4.3 ± 0.04	4.3; 4.2–4.5	4.3 ± 0.04	4.3; 4.2–4.5	<0.001
Ionized calcium, mmol/L	1.4 ± 0.01	1.4; 1.4–1.5	1.3 ± 0.01	1.3; 1.2–1.3	<0.001
Glucose, mmol/L	89.9 ± 2.8	89.5; 82.3–97.0	98.3 ± 2.8	100; 93.3–105.0	<0.001
Hematocrit, %	43.1 ± 1.0	42; 40–47	45.5 ± 1.0	45; 43–48	<0.001
Hemoglobin, g/dl	14.7 ± 0.41	14; 13.6–16.1	15.5 ± 0.41	15.3; 14.6–16.3	<0.001

### Factors Related to Stamina

When controlling for breed, the following independent variables: outdoor temperature (*p* = 0.005), pre-exercise activity (*p*=0.008), post-exercise activity (*p* < 0.001), reduction in BEecf (*p* = 0.044), and decrease in TCO_2_ (*p* = 0.005) were all significantly related to total activity (Table 5). The following independent variables were tested in this model, but were dropped from the model; age, sex, outdoor humidity, left ear temperature, right ear temperature, PR, lactate, venous blood pH, gases (PvCO_2_, HCO_3_, PvO_2_, SvO_2_), electrolytes (iCa, Na, K), glucose, hemoglobin, and hematocrit. A two-way ANOVA was performed with stamina as the dependent variable. When controlling for breed, stamina was not influenced if the dog was affected by tracheobronchitis (*p* = 0.67).

**Table 5:** Regression coefficients of multiple regression for independent variables associated with total activity.

	Coefficient	*p*-Value
Dog breed	−94.081	0.008
Outdoor temperature (°C/°F)	−23.824	0.005
Pre-exercise total activity	0.149	0.008
Post-exercise total activity	0.616	<0.001
Reduction in Base excess in extracellular fluid compartment	69.325	0.044
Reduction in TCO_2_	−62.995	0.005

### Factors Related to Change-from-Baseline CBT

When controlling for breed and sex, regression analysis was performed with CBT as the dependent variable. Study day (*p* = 0.005), increase in PR (*p* < 0.001), increase in lactate (*p* = 0.001), reduction in BEecf (*p* = 0.031), increase in glucose (*p* = 0.044), increase in hematocrit (*p* = 0.032), and increase in hemoglobin (*p* = 0.038) were all significantly related to an increase in CBT.

### Interaction of Time, Day, and Time × Day

A linear mixed model was run where Time, Day, and Time × Day were entered in as fixed effects and animal ID was entered as a random effect. This accounts for the repeated nature of the data. The effect of study day was significant for sodium (Na) on Day −4 vs. Day 3 (*p* = 0.04), and Day 3 vs. Day 11 (*p* = 0.05), and for hemoglobin on Day −4 vs. Day 15 (*p* = 0.03), Day 3 vs. Day 15 (*p* = 0.01), and Day 8 vs. Day 15 (*p* = 0.039). For the interaction of Time × Day, there was a significant difference for lactate on Day 8 vs. Day 3 pre-exercise (*p* = 0.047), for Na on Day 3 vs. Day −4 post-exercise (*p* = 0.04), for potassium on Day 11 vs. Day 3 post-exercise (*p* = 0.04), for PR on Day 15 vs. Day −4 post-exercise (*p* = 0.49), for pH on Day 15 vs. Day −4 post-exercise (*p* = 0.03), for Glu on Day 15 vs. Day −4 post-exercise (*p* = 0.028), for Hb on Day 15 vs. Day −4 post-exercise (*p* = 0.035), for pH on Day 15 vs. Day 3 post-exercise (*p* = 0.02), for Hb on Day 15 vs. Day 3 post-exercise (*p* = 0.007), for CBT on Day 15 vs. Day 8 post-exercise (*p* = 0.04), for pH on Day 15 vs. Day 8 post-exercise (*p* = 0.04), for Hb on Day 15 vs. Day 8 post-exercise (*p* = 0.013), for SvO_2_ on Day 15 vs. Day 11 post-exercise (*p* = 0.039), for Glu on Day 15 vs. Day 11 post-exercise (*p* = 0.036), and for Hb on Day 15 vs. Day 11 post-exercise (*p* = 0.013) (Table 6).

**Table 6:** Linear mixed model of interactions between Time, Day, and Time × Day.

	Pre-exercise (mean ± SE)	Post-exercise (mean ± SE)	*p*-Value (pre vs. post)	*p*-Value (Day)	*p*-Value (Time × Day)
Core body temperature, °C/°F	38.3 ± 0.3/101.0 ± 0.3	40.1 ± 0.1/105.4 ± 0.3	<0.001		PE 8 vs. PE 15 *p* = 0.04
Left ear temperature, °C/°F	37.3 ± 0.1/98.8 ± 0.4	39.8 ± 0.1/103.7 ± 0.4	<0.001		
Right ear temperature, °C/°F	37.2 ± 0.2/98.9 ± 0.4	39.9 ± 0.1/103.9 ± 0.3	<0.001		
Pulse rate, beats/min	86.9 ± 3.3	134.6 ± 3.3	<0.001		PE 4 vs. PE 15 *p* = 0.49
Lactate reading, mmol/L	1.2 ± 0.09	1.4 ± 0.09	0.007		Pre 3 vs. Pre 8 *p* = 0.04
pH	7.37 ± 0.02	7.56 ± 0.02	<0.001		PE −4 vs. PE 15 *p* = 0.03
					PE 3 vs. PE 15 *p* = 0.02
					PE 8 vs. PE 15 *p* = 0.04
PvCO_2_, mmHg	37.5 ± 1.06	20.0 ± 1.06	<0.001		
PvO_2_, mmHg	45.4 ± 4.03	70.4 ± 4.03	<0.001		
Base excess in extracellular fluid compartment, mmHg	−3.6 ± 0.48	−4.8 ± 0.48	<0.001		
HCO_3_, mmHg	21.5 ± 0.43	17.3 ± 0.43	<0.001		
TCO_2_, mmHg	22.8 ± 0.47	17.8 ± 0.47	0.001		
SvO_2_ %	74.0 ± 2.2	92.4 ± 2.2	<0.001		PE 11 vs. PE 15 *p* = 0.04
Sodium, mmol/L	145.2 ± 0.32	145.0 ± 0.32	0.39	Day −4 vs. Day 3 *p* = 0.04	PE −4 vs. PE 3 *p* = 0.04
				Day 3 vs. Day 11 *p* = 0.05	
Potassium, mmol/L	4.3 ± 0.04	4.3 ± 0.04	0.38		PE 3 vs. PE 11 *p* = 0.04
Ionized calcium, mmol/L	1.4 ± 0.01	1.3 ± 0.01	<0.001		
Glucose, mmol/L	89.9 ± 2.8	98.3 ± 2.8	<0.001		PE −4 vs. PE 15 *p* = 0.03
					PE 11 vs. PE 15 *p* = 0.04
Hematocrit, %	43.1 ± 1.0	45.5 ± 1.0	<0.001		
Hemoglobin, g/dL	14.7 ± 0.41	15.5 ± 0.41	0.57	Day −4 vs. Day 15 *p* = 0.03	PE −4 vs. PE 15 *p* = 0.04
				Day 3 vs. Day 15 *p* = 0.01	PE 3 vs. PE 15 *p* = 0.01
				Day 8 vs. Day 15 *p* = 0.039	PE 8 vs. PE 15 *p* = 0.01
					PE 11 vs. PE 15 *p* = 0.01

*For the interaction of Time × Day, pre- vs. post-exercise on any given day was significant for all values except sodium (Na) and potassium (K). For the interaction of Day, only Na and hemoglobin were significant, all other values did not show a significant interaction*.

## Discussion

This study evaluated the environmental and dog-specific variables that influence exercise stamina in regularly exercised working dogs. Stamina was defined as a two-component assessment of activity, as measured by an accelerometer and duration of exercise. Selection of working dogs can be based on specific parameters that include size, conformation, gait during exercise, past and current orthopedic history, body condition score, fitness level, and behavior (31). However, environmental and dog-specific factors that are associated with stamina have yet to be studied. This study uniquely shows that pre-exercise activity, post-exercise activity, outdoor temperature, reduction in both BEecf and TCO_2_ are factors related to stamina.

The exercise challenge used in this study resulted in an increase in body temperature, lactic acidosis, respiratory alkalosis, and hypocapnia. The regression model defining factors associated with stamina included two biomarkers of alkalosis; BEecf and TCO_2_, as stamina increased, BEecf and TCO_2_ decreased. These findings are consistent with previous studies investigating the effect of intense, short-term exercise on canine hematology and physiology (2–4, 6, 7, 24, 28, 32). In contrast to endurance, this study of stamina found significant respiratory alkalosis with pH as high as 7.89 and an associated increase in PvO_2_ and decrease in ${\text{HCO}}_{3}^{-}$. Glucose and hematocrit also increased significantly. An increase in glucose after short-term exercise has been reported in other studies (4, 7). Increased hematocrit is commonly associated with acute exercise, rather than endurance exercise. Similar to other studies (33, 34), strenuous exercise has been shown to cause an increase in panting resulting in a loss of PvCO_2_ and a decrease in ${\text{HCO}}_{3}^{-}$. The exercise challenge used in this study demonstrated metabolic changes associated with intense exercise in high ambient temperatures. These changes are associated with limited stamina; therefore, strategies to enhance stamina will need to consider these physiologic factors.

The data reported above indicate that outdoor temperature, but not humidity, was the environmental variable that remained in the model, and inversely related to predicting stamina; as outdoor temperature increased, stamina decreased. Others have observed that high ambient temperatures acutely induce metabolic changes in exercising dogs, which is consistent with the current study’s results (4, 6). Although humidity was significantly different between the two exercise time periods, this factor did not have an impact on stamina.

Since hyperthermia can limit performance and result in serious medical consequences, the identification of predictors of increased CBT could provide useful information for proper health care and management of exercising dogs. Dogs in this study had post-exercise CBTs ranging from 38.9 to 42.4°C/102–108.3°F (average 40.1°C/105.4°F), LETs ranging from 36.3 to 42.1°C/97.4–107.7°F (average 39.8°C/103.8°F), and RETs ranging from 35.6 to 42.4°C/96–108.4°F (average 39.9°C/103.9°F). An increase in CBT and PR leads to an increase in panting, resulting in a loss of pCO_2_. Dogs are capable of increasing its carotid blood flow by 500% and cardiac output by 74–200% during exercise, conceivably to cope with the energy demands required for thermoregulation. The fixed factors of dog breed, sex, and age require further study in order to better understand thermoregulatory differences, particularly in light of the low numbers of certain breeds in this study. The independent variables included in the model that relate to an increase in CBT were the metabolic factors of increase in PR, lactate, glucose, hematocrit, and hemoglobin, as well as decrease in BEecf, which is consistent with the relationship of exercise-induced changes in hematological values in similar studies investigating the physiological effect of short-term strenuous exercise on dogs (2, 4, 7, 24, 35). Splenic contraction increases during brief exercise resulting in increased hematocrit levels. As hematocrit increases, oxygen carrying capacity increases driving increased oxygen delivery. Consistent with Steiss and Wright (4), there was a significant increase in glucose after exercise. An increase in blood glucose is associated with a physiological stress response coupled with increased exercise. An increase in BEecf is a consequence of increased panting, and finally an increase in PR is an expected outcome of increased activity and the associated increase in CBT (36, 37).

When controlling for breed and sex, the fixed factors that remained in the model predicting increase in CBT was study day. The inclusion of study day (absolute study day in 19-day exercise period) in the model suggests that dogs have the capability of acclimation to consistent exercise in hot ambient temperatures over 19 days; however, temperature and humidity did not remain in the model predicting increase in CBT, suggesting absolute temperature average 28.7°C/83.7°F and humidity average 49.6% do not play a significant role affecting thermoregulation during exercise in dogs. Heat acclimation has not been well studied in working dogs, and further investigation is required to fully understand this mechanism.

Pre-exercise activity and post-exercise activity were both included in our model as predictors of stamina. Dogs with higher pre-exercise activity demonstrated higher stamina during exercise. In addition, higher post-exercise activity was associated with higher stamina during exercise. An overlooked yet fundamental aspect of exercise training for a working dog is a pre-exercise warm up and a post-exercise cooldown (3). “The warm-up mildly increases the ambient temperature of muscles and joints, and lubricates fascia to reduce risk of injury, susceptibility to trauma, as well as allowing for a greater stretch” (38). “The increased musculoskeletal temperature causes local vasodilation, shifts the oxy-hemoglobin dissociation curve, and increases muscles contraction and relaxation speeds” (39, 40). One type of warm up for human, horses, and dogs, consists of 15 min exercise intensity that increase heart rate by about 70% (41). The cool-down exercises are low intensity and ensure that blood continues to circulate from the muscles to wash out the waste products of muscle metabolism (42). Pre-exercise activity could reflect warm up activity and post-exercise activity could reflect cooldown activity or this relationship can be explained by dogs that are active are active across all time periods (sometimes referred to as the behavior of high “drive”/motivation) have higher stamina. More research is required to clarify the impact of warm-up/cool-down on stamina. Although not studied here, increased warm-up/cool-down activity may lead to fewer injuries and less medical intervention for dogs during strenuous exercise.

A few dogs used in the current study demonstrated elevated CBTs as high as 42.4°C (108.3°F) with exercise. CBT above 40.6°C (105°F) has been historically defined as heat stroke (43). However, no dogs participating in this study experienced any of the clinical manifestations of heat stroke or heat injury. Our observations were similar to other reports of working dogs (3, 4). The dogs in the current study were regularly trained to work in a hot environment, thus were accustomed to exercising with increased CBTs. In addition, CBT recovered rapidly without intervention (unpublished data). For pet dogs not accustomed to training and conditioning in a warm climate, it is ideal for exercise to occur during the cooler part of the day to minimize exposure to warmer temperatures. More importantly, communication regarding the recognition of these signs to pet owners should aid in their awareness of the pet’s condition when a thermometer is likely to not be readily available.

There were several important limitations in this study. The sample population was small and included all young, healthy dogs with experience in agility and search exercises, and accustomed to strenuous activity in high ambient temperatures. Although the study population only accounted for five different breeds with unequal representation in each breed, this independent variable of breed remained in the model as a significant factor associated with stamina, requiring the need of further investigation into the effect of breed and its relation to canine exercise. Two dogs did not regularly complete the ball-retrieve exercise and instead were trotted in a ball-retrieve fashion, which may have introduced a bias. Unlike in humans where fitness can be defined by VO_2max_, there was no readily available quantitative measure of fitness in these dogs. Further investigation into the relationship between fitness status and stamina would be beneficial to understanding the cardiovascular and respiratory systems of working dogs. The order of dogs being tested was randomized unless medical reasons dictated otherwise. Several dogs were affected by tracheobronchitis, and on a separate occasion, one dog suffered from an abrasion on the lower left hind limb (did not occur as a result of the current study) and was rested one day before being tested again. Additionally, the thermometer used for measuring left and right ear temperatures tended to be sensitive to the user and positioning of the probe, resulting in measurements that were lower than expected. Finally, the interactions of Time, Day, and Time × Day were statistically significant, but not clinically significant. Day 15 had the most significant interactions with other study days; however, the Day 15 post-exercise average of total activity (1,777.2 count/min) and outdoor temperature (82.3°F) was lower than the average total activity and outdoor temperature of the study. This frequency of Day 15 interaction significance may be due to an accumulation effect of consistent exercise throughout the study period resulting in muscle soreness or fatigue.

In conclusion, when controlling for breed, increase in pre-exercise and post-exercise activity, high outdoor temperature, and respiratory alkalosis were significantly related to stamina. Working dogs are examples of superior athletes that can withstand working in hot ambient temperatures for short periods of time that include exercising with elevated CBTs as high as 42.4°C (108.3°F), pH values reaching 7.89, and PvCO_2_ levels as low as 8.4 mmHg. Physical signs associated with exercise in hot ambient temperatures were used to terminate exercise in order to prevent heat injury. To evaluate relevant parameters for common working dogs (search, law enforcement, etc.), activity and duration of exercise were used to define stamina. Surprisingly, CBT did not influence stamina, rather respiratory efficiency or ability to eliminate heat without creating profound acid/base disturbances was a major factor influencing stamina. This study not only defined canine stamina, but investigated the physiological, hematological, respiratory, and cardiovascular factors, fixed factors such as age, sex, breed, and environmental factors of outdoor temperature and humidity, that are associated with stamina during exercise in high ambient temperatures.

## List of Approvals

### Data Set Information

Name of data set: predictors of stamina in dogs exercising in high ambient temperatures
Name of database/repository: FigShare
Url link: https://figshare.com/articles/Stamina Manuscript Data_xlsx/3581457

The data collection period was from June 12, 2014 to August 27, 2014. Data were acquired using the methods listed in the Section “Materials and Methods.” No filters were applied to the data set. Readers are able to use this data set in any capacity they may choose.

## Ethics Statement

This study was carried out in accordance with the recommendations of Animal Welfare Act and the University of Pennsylvania, Institutional Animal Care and Use Committee. The protocol was approved by the University of Pennsylvania Institutional Animal Care and Use Committee protocol (805342) on 6/5/2014.

## Author Contributions

PR led the study data collection, participated in study design, data analysis, and manuscript preparation. MR participated in data collection and manuscript review. BZ participated in study design, data review, and manuscript review. CO oversaw the design of the study, assisted with data collection, and performed data analysis and manuscript preparation.

## Conflict of Interest Statement

The study was funded by Nestle Purina Research. The final manuscript was reviewed by Nestle Purina. The manuscript does not contain any commercial product or potential for conflict of interest.
